# Social–Structural Antecedents Come Forward to Elicit Envy to Distant Out-Groups

**DOI:** 10.3389/fpsyg.2021.610571

**Published:** 2021-05-31

**Authors:** Nino Javakhishvili, Nino Butsashvili, Irina Vardanashvili, Anna Gogibedashvili

**Affiliations:** Dimitry Uznadze Institute of Psychology, Ilia State University, Tbilisi, Georgia

**Keywords:** socio–structural antecedents, stereotypes, emotions, single group design, mediation and moderation

## Abstract

This study utilizing correlation, regression, confirmatory factor analyses (CFAs), ANOVA, moderation and mediation analysis investigated connections of stereotypes, emotions, and sociocultural variables in a single-sample/single-group design. Prior to data processing, Georgian versions of the Stereotype Content Model (SCM) questionnaires were validated through CFA. The study looked at Georgian students' attitudes to: (a) representatives of German-speaking countries (87 participants) and (b) representatives of English-speaking countries (244 participants). Emotions predicted to these groups by social–structural antecedents—vitality and fear of assimilation—and stereotypes were admiration, pride, and sympathy. In addition, envy was predicted for the English-speaking group. The prediction of envy is explained by moderation analysis according to which it is elicited by the interplay of warmth and competence, as well as fear of assimilation and competence. The former interaction mediates the link between social–structural antecedents to emotions. Thus, distant out-groups elicit envy as a result of their perceived vitality, fear of assimilation, warmth, and competence. Social–structural antecedents come forward to elicit emotions of envy independently as well as in interaction with stereotypes when small country representatives evaluate representatives of the influential group of English-speaking people.

## Introduction

The Stereotype Content Model (SCM) has been proven to work panculturally (Cuddy et al., 2009; Durante et al., 2013; Fiske, 2015). It was developed and tested first in the United States and then in Western Europe, Asia, Eastern Europe, and the post-Soviet space. The latter showed some cultural differences that were explained by the context, namely, the socialist arrangement of societies studied (Grigoryan et al., 2019).

The wide international usage of the SCM speaks of the strength and robustness of the theory, which is supported cross-culturally by rich data from multiple in-groups and out-groups assessed. The initial focus of the model, hence, the title, is on two basic stereotypes of competence and warmth, which in combination with each other forms four possible quadrants or clusters. If a group and its representatives are perceived as highly competent and warm, they belong to the HC-HW cluster, which, according to numerous data, are mostly in-groups; if a group is perceived as deserving low competence and low warmth, it belongs to the LC-LW cluster with mostly avoided out-groups, such as the homeless. These two clusters are univalent, but the other two are ambivalent, with either competence or warmth being substantially higher than the other. The ambivalent cluster of HC-LW is usually rich people, while the LC-HW cluster is usually the elderly (Cuddy et al., 2008; Fiske, 2018).

In a relatively later development of the model, stereotypes are combined to elicit the corresponding emotions: the combination of warmth and competence elicits admiration and pride if both are high, disgust, and contempt if both are low, pity, and sympathy if warmth is high but competence is low, and envy and jealousy if competence is high but warmth is low (Fiske et al., 2002; Cuddy et al., 2007; Fiske, 2018). Usually, scholars group the pairs of emotions and use average scores in the analysis. Thus, admiration and pride go under admiration; disgust, contempt, resentment, and anger go under disgust; pity and sympathy go under sympathy, while envy and jealousy go under envy (Fiske et al., 2002; Cuddy et al., 2007).

Later, the authors of the SCM have investigated mediational chains starting with the stereotypes through emotional prejudice to the corresponding behaviors. These are called Behaviors from Intergroup Affect and Stereotypes—the BIAS map, e.g., from competence to the corresponding behavior of passive facilitation (cooperation and association) through the corresponding emotions of admiration and envy and from lack of warmth to the corresponding behavior of active harm (fight, attack) through the corresponding emotion of contempt and envy (Cuddy et al., 2007; Becker and Asbrock, 2012; Ufkes et al., 2012). Later, these findings were replicated in the Norwegian sample but with only one emotion mediating the links between stereotypes and corresponding behaviors (e.g., the path between competence to passive facilitation is mediated by envy; Bye and Herrebrøden, 2018). Paths from stereotypes to harmful behaviors through emotions of anger and fear were found in the study of prejudice toward the mentally ill (Sadler et al., 2015). Not exactly the BIAS map but a similar chain was demonstrated by the British psychologists *via* path analysis from competence to help through pity or through admiration (study 2) (Sweetman et al., 2013).

Structural antecedents of stereotypes—competitiveness and status of the groups in a society—represent the initial focus of the SCM. If groups are perceived as having a high status, they are stereotyped as competent, and if groups are perceived as competitive, they are perceived as cold. According to SCM, in its classical understanding, perceived status is rather linked with competence than with warmth, and perceived competition is rather linked with warmth than competence (Fiske et al., 1999; Glick et al., 2006; Caprariello et al., 2009). Some of the further studies, however, detected the diagonal links as well: status is also linked with warmth, and competition is also linked with competence (Fiske et al., 2002; Durante, 2008; Tsukamoto and Fiske, 2018; Froehlich and Schulte, 2019). As for the path from the structural antecedents to emotions, Glick et al. (2006) found correlations among social structural variables, stereotypes, and emotions. In the 2015 study of Singaporeans, the authors, using regression analysis, found that both realistic and symbolic threats considered as competitiveness predicted prejudiced emotions to four out-groups, while competence and warmth scores did not (Ramsay and Pang, 2017). Caprariello et al. (2009) demonstrated such links, including interaction of social–structural variables to elicit emotions.

Stereotypes and their antecedents have been studied about various groups residing within a country listed by the sample of the country representatives, such as the elderly, Christians, Muslims, students, the homeless, etc. As in many other studies of prejudice, distant groups not residing in the same country were not of much interest to the SCM. However, considering globalization, thanks to which people from all over the world interact either in person or virtually, using the World Wide Web, and communicate with tourists or business partners from very distant parts of the world, the need to study prejudice to the representatives of “distant out-groups” may also become a focus of the SCM. Indeed, in 2006, Glick et al. (2006) studied attitudes of Latin Americans, Europeans, Asians, and Australians to the North Americans and found that the social–structural antecedents correlate with corresponding stereotypes and emotions. In 1997, in the frames of a different theory, which is compatible with the SCM (Kervyn et al., 2010), Phalet and Poppe (1997) studied stereotypes and their antecedents in six Eastern European samples (two of them being from the former Soviet Union) to Germans, English, and Italians. As more than 20 years have passed since then and relations on the international arena have changed, the current study aims to present the most recent picture of how small country representatives (like Georgians) view English and German language speakers, including not only western Europeans but also Americans and other large country representatives from different continents.

This article presents findings from one of the former Soviet Union republics from the South Caucasus, Georgia, studies from which are underrepresented among the international community of professionals worldwide. Investigation of this space might bring interesting findings that will enrich already accumulated knowledge on the SCM.

Georgia is a still young independent state with an underdeveloped economy and a hybrid democratic regime. The majority of the population of this former Soviet republic has long aspired toward the West. Soon after the country regained independence, this wish turned into an officially declared aim of the country to join the European Union (Gvalia et al., 2013; Georgian Center for Security Development, 2017). Thus, attitudes of Georgians to out-groups from the EU as well as the USA, a major supporter of Georgia's democracy and economy, came into focus of public opinion polls and social research (Mestvirishvili and Mestvirishvili, 2014; Caucasus Research Resource Centers, 2017, 2019; International Republican Institute, 2018). These studies unequivocally show that attitudes toward Europeans are positive. Some of them used a widespread prejudice measure of social distance, which is considered a behavioral aspect of prejudice (Javakhishvili et al., 2012, 2018; Caucasus Research Resource Centers, 2017, 2019; National Democratic Institute, 2019). Initially, the scale was used to measure social distances to immigrants living in the USA; however, other studies included out-groups residing outside of the country investigated (Thyne and Lawson, 2004; Sinkovics and Penz, 2009). Such interests were fostered by emerging globalization, new business relations, and the development of the tourism industry. This is especially true about Georgian students who, unlike their parents and grandparents, travel abroad and host international tourists as well as communicate over the Internet.

A Soviet republic for 70 years (1921–1991), Georgia was behind the iron curtain for the entire period, with Russian being the only foreign language for the vast majority of its population. Russian gradually expanded as the main language of communication over the extensive area of the USSR, resulting in mass bilingualism by the 1990s. It has to be noted that Georgian language is totally different from Russian as well as from European languages. It belongs to the group of Caucasian languages but is spoken and understood only by Georgians. It also has a unique alphabet, with the earliest surviving inscription dating from the 4th century BC. In the Soviet times, Russian was taught at schools from the first years of study, while European languages, mostly French, English, and German, were taught from the fifth year of study. The quality of learning European languages was much lower than that of native, Georgian, and Russian, as the former were not used in either daily or professional communication. At the same time, from the 1970s, many Georgians became interested in the West, and after the Soviet Union breakup, many young people went to Germany, UK, and USA to receive higher education. Currently, Russian is spoken by the older generation, while young people speak English or German or other European languages. Now that Georgia has declared its willingness to join the European Union, learning respective languages has become even more relevant.

Starting from 1996, three studies measured students' social distances to 22 out-groups where the data obtained from the modified Bogardus social distance scale showed that representatives of Western European countries and the USA were placed on the top of the list in all cases (Javakhishvili, 2005; Javakhishvili et al., 2012). The authors explained such results by soft and hard power of these societies in the eyes of Georgian students, who characterized them as having democratic values, good education, and strong economies (Javakhishvili et al., 2018).

This time, we aimed to demonstrate that the SCM approach and measure could yield more precise and concise information as to why these groups are held so close by Georgian students. Specifically, in the current study, we aimed to show how Georgian students perceive the representatives of these out-groups based on their characteristics on the international arena and what they feel toward them.

## The Present Study

In the present study, we examined the new context and used the different methodological approaches to show what happens when small country representatives evaluate representatives of large countries. The umbrella question of the current paper is: do vitality and fear of assimilation produce the stereotypes of competence and warmth, which, in turn, produce emotional consequences? And how?

To address this question, we used a single-sample/single-group design; therefore, we utilized some methodological approaches rarely applied in the studies of SCM. Some scholars who investigate SCM and related variables proposed a number of approaches to data processing, such as using regression to find more precise links between structural antecedents and stereotypes (Durante et al., 2013; Kervyn et al., 2015; Grigoryan et al., 2019; Grigoryev et al., 2019). Some authors went farther to use path analysis as a more comprehensive way to analyze predictions (Froehlich and Schulte, 2019), while others propose to process data on a latent, rather than observed, level—for example, calculate latent means (Kotzur et al., 2018, 2019). These new approaches will, inevitably, be used more and more frequently, while in the present study, we use some components of path analysis—regression analysis to check moderations and mediations using the PROCESS macro developed by Andrew Hayes (Process macro version 3.5 developed for SPSS by Hayes, 2017), which simplifies our work, as it produces outputs of conditional effects and their graphic display, as well as standardized coefficients of predictors, and enables mean centering variables in interaction.

First of all, we have analyzed emotions separately, not to “mask variability” and to bring more information to the analysis of emotions and their relation with stereotypes. Separately considered emotions would enable us to better comprehend the “textured nature of intergroup relations” (Matthews and Levin, 2012, p. 2). We will proceed farther to examine if warmth and competence elicit corresponding emotions not only in combination but in interaction *via* moderation analysis. This approach has been tested in two studies (Sweetman et al., 2013; Kotzur et al., 2018), resulting positively in the first but negatively in the second case. As a result of such inconsistent findings, Tsukamoto and Fiske (2018) advise to investigate the interaction of warmth and competence in future studies. Indeed, moderation analysis will help us better understand which emotions are elicited by stereotypes.

Secondly, we investigated mediational chains, similar to BIAS map, but from social–structural indicators through the corresponding stereotypes to the corresponding emotions. To put this aim in the SCM terminology: how structural antecedents status and competitiveness of English- and German-speaking groups trigger perceived stereotypes—warmth and competence—which, in turn, trigger corresponding emotions. This alignment of antecedents, stereotypes, and emotions in a mediational chain as proposed by Cuddy et al. (2007) has not been tested yet and will bring an added value to the SCM theory. At the same time, with such an approach, we demonstrate the role of the SCM framework beyond the traditional measures of prejudice.

Thirdly, we measured status and competition by other variables, such as vitality and fear of assimilation. The latter is closely connected to threat, which coincides with competition (Fiske et al., 1999; Caprariello et al., 2009); it also speaks about the respondents' group, in our case, representatives of Georgia, who might be afraid to lose their own culture and language as a result of globalization. Indeed, the questions on realistic and symbolic threat were entered into the SCM survey (Kervyn et al., 2015). Some questions about status and competition would not be compatible with the groups we studied, so vitality and fear of assimilation were deemed more appropriate. For example, a question on status, “how prestigious are the jobs of the representatives of this group—are,” is feasible when assessing groups that reside in a country, not outside, as it was in our case. The fear of assimilation questionnaire contains the term “threat” in two questions out of the total three, which, according to the Integrated Threat Theory (ITT), can be understood as tapping into symbolic threat. However, our survey does not measure realistic threat, which is also covered by ITT (Stephan et al., 2009). Vitality can be considered a proxy of status to the extent that we asked our participants how developed German- and English-speaking cultures are and if they play an important role in the world.

Thus, in the present study, we address the issue by investigating English- and German-speaking groups. We examined direct links from antecedents to stereotypes and emotions; also, we went one step further to examine interactions of status/competition with warmth/competence scores to predict emotions. Such interactions, which to the best of our knowledge have not been studied so far, enable us to see deeper into certain emotions elicited.

Considering the roles of the English- and German-speaking countries on the international arena and for Georgia, we assumed that in the eyes of our participants, German- and English-speaking group representatives appear as vital but posing relatively less symbolic threat. Respectively, their perceived competence and warmth would be high. These combinations end up in the respective emotions as provided in the SCM. Accordingly, we hypothesized that:

1. Vitality scores would be higher than fear of assimilation scores for both English- and German-speaking groups;
2. Competence scores would be higher than warmth scores for both English- and German-speaking groups;
3. The German- and English-speaking groups will produce higher scores on the emotions of admiration and pride than for the rest of them.

As this is a correlational study, we applied regression analysis as noted above to study links among the three components of the SCM. Hence, we had the following hypotheses:

4. Vitality and fear of assimilation predict corresponding stereotypes independently as well as in interaction with each other;
5. Vitality, fear of assimilation, warmth, and competence predict corresponding emotions independently as well as in interaction with each other;
6. Warmth and competence mediate links between vitality and fear of assimilation and corresponding emotions;
7. Interaction of warmth and competence mediate the link between vitality and fear of assimilation and corresponding emotions.

The study investigates attitudes of Georgian undergraduate students toward the representatives of German- and English-speaking people. Study a. examines attitudes toward German language speakers and study b. toward English language speakers. The criterion for participation was learning of English and/or German. Since English as a second language is compulsory at Georgian universities, any undergraduate student would meet our criteria, which is not the case with German—we had to find out if any of the students was a German language learner as well. Using these two groups would help us understand what Georgians think about geographically distant but still very familiar groups, as many Georgians, especially the younger generation, are interested in their culture (as mentioned above).

We used a single-sample/single-group design, thus providing individual-level analysis of data. For this reason, we compared mean scores of the nine emotions as well as conducted regression analysis to see which of these emotions are predicted by competence and warmth scores as well as their interaction. In addition, we regressed emotions on status and competition scores to investigate their role in predicting emotions, as well as the role of their interaction with each other and stereotypes. Prior to these, we had to define whether the original scale of stereotypes maintains the same two-factorial structure of competence and warmth in its Georgian version.

## Methods

### Participants and Procedure

We recruited two samples: study a.−87 participants who were studying German as their second language, while Georgian is their native tongue. Their age varied between 18 and 34 (mean age 21.54, *SD* = 2.78). Most of the participants were females, 76.2%; and study b.−240 respondents who were studying English as their second language, while Georgian is their native tongue. Age range was 18–36 (mean age 20.62, *SD* = 2.49). Most of the participants were females, 76.6%. All of the participants were students from various universities in Tbilisi, the capital of Georgia.

The participants filled out a self-administered survey after providing informed consent. The survey was conducted partially online and partially in a paper-pencil mode. We contacted English and German language teachers and asked them to inform their students about our research. This questionnaire did not include personal identification data, and the ethical standards were closely followed. The respondents' anonymity was guaranteed, and all of them were informed that they could stop participating any time, without submitting answers.

### Measures

#### Stereotypes

To measure stereotypes, we used a modified questionnaire from the study of Cuddy et al. (2007). The questionnaire was translated into Georgian for another international study (the data file can be accessed at https://osf.io/w2mbz/; see also Grigoryan et al., 2019). The scale contained eight questions of stereotypes—three of warmth, five of competence. The questions were answered on a 5-point Likert-type scale.

The respondents answered questions on what “Most Georgians” or “People” think about English and German speakers, as provided in the original scale of Cuddy et al. (2007). For example, “To what extent do most Georgians view English speakers as warm?” We used a 5-point Likert scale, where “1” meant “not at all”; “5” meant “extremely.”

#### Emotions

To measure emotions felt toward German- and English-speaking groups, we used the same questionnaire. The scale assessed nine emotions: admiration, pride, sympathy, pity, envy, anger, resentment, contempt, and disgust. The English version of the scale contained 10 items, but “jealousy” was removed from the Georgian questionnaire due to the translation problem—no appropriate word in the Georgian language was found to cover its meaning.

As above, the respondents answered questions on how “Most Georgians” or “People” feel toward English and German speakers. A sample item is “To what extent do people tend to feel pity toward English speakers?

#### Fear of Assimilation

We used the Fear of assimilation scale to study the respondents' attitude toward globalization and its effect on the local culture. Globalization can be considered a symbolic threat toward one's own beliefs and traditions, making the mainstream culture as a competitor. The scale contained three items and was modified from the original version in the study by Ryan (2008). An example of the items is “As globalization advances, there is a danger of losing the Georgian language and culture.”

#### Vitality

A four-item Vitality scale (Ryan, 2008) was used to measure the participants' estimation of the importance of English- or German-speaking countries. A questions sample is “Do you think that English-speaking countries have an important role in the world?” For all questions, we used the five-point Likert scale, where “1” meant “not at all” and “5” meant “extremely.”

## Results

### Confirmatory Factor Analysis/Validation

Before proceeding with testing the hypotheses, we first examined the factorial structure of the competence/warmth scale. The competence/warmth scale was translated into Georgian and then back-translated. These translations were additionally analyzed by a team of experts (psychologists and linguists). The Georgian version of the competence/warmth scale was validated *via* confirmatory factor analysis (CFA) in MPlus, version 6.12. Data from both a. and b. studies were merged into one file to increase data size. We checked the model for two factors: competence and warmth. The model fit indices were all good: χ^2^ = 57.04, *p* < 0.001, root mean square error of approximation (RMSEA) = 0.08, comparative fit index (CFI) = 0.94, Tucker–Lewis index (TLI) = 0.91, standardized root mean square residual (SRMR) = 0.05, as were factor loadings of items on competence and warmth subscales. The rest of the analysis was conducted on 2a and 2b data separately. After finding that the Georgian version of the SCM scale provides the same two factorial structures of stereotypes as in the original version, we proceeded with answering questions of the present study. To address multiple comparison problems, we applied false discovery rate (FDR) technique to correlation, regression, moderation, and mediation analyses. This technique adjusts *p*-values *via* applying q = 0.01 threshold, so that, for example, former *p*-value of 0.02 might become 0.04 or higher than 0.05 (McDonald, 2014). As a result, only the adjusted *p*-values are reported below.

### Hypothesis Testing

The first three hypotheses are addressed below separately for the German- and English-speaking groups.

#### German-Speaking Group

To answer hypothesis 1, we calculated mean scores of the German-speaking group for vitality and fear of assimilation and compared them by paired samples *t*-test, which showed a significant difference: *M* = 4.42, *SD* = 0.53 for vitality and *M* = 2.42, *SD* = 1.03 for fear of assimilation; *t*_(86)_ = 15.98, *p* < 0.001. There was a non-significant correlation between these two variables.

To answer hypothesis 2, we calculated mean scores of the German-speaking group for competence and warmth and compared these with each other. The within subjects/paired samples *t*-test showed that competence scores were higher than those of warmth: *M* = 4.26, *SD* = 0.61 for competence and *M* = 3.40, *SD* = 0.06 for warmth; *t*_(86)_ = 8.93, *p* < 0.001. These two stereotypes are moderately correlated: *r* = 0.37; *p* < 0.001.

To address hypothesis 3, we calculated German-speaking group emotion scores separately for each and used ANOVA to compare these. Table 1 below provides the data obtained.

**Table 1:** Mean score of emotions for the German-speaking group.

	**Admiration**	**Sympathy**	**Pride**	**Envy**	**Anger**	**Pity**	**Resentment**	**Contempt**	**Disgust**
German-speaking group	**3.56 (1.18)**	**3.05 (0.99)**	**3.01 (0.99)**	2.99 (1.23)	2.15 (1.03)	2.14 (1.13)	2.09 (1.08)	1.96 (1.12)	1.82 (0.94)

*Standard deviations are shown in parentheses*.

*Scores that significantly differ from the other scores are provided in bold*.

Besides emotions of admiration and pride, sympathy also deserved a high score. ANOVA shows that the mean score for admiration significantly differs from all other scores, *F*_(8,616)_ = 30.31, *p* < 0.001, while, according to pairwise comparisons, pride and sympathy are not significantly different from each other, *p* > 0.05, and in all cases, even envy is not significantly different from pride.

To sum up, for the German-speaking group, hypotheses 1 and 2 are confirmed, while hypothesis 3 was partially confirmed, as sympathy gained high scores in addition to admiration and pride.

#### English-Speaking Group

To answer hypothesis 1, we calculated mean scores for vitality and fear of assimilation and compared them by paired samples *t*-test: *M* = 4.20, *SD* = 0.54 for vitality and *M* = 2.46, *SD* = 0.91 for fear of assimilation; *t*_(239)_ = 24.71, *p* < 0.001, showing a significant difference. These two variables did not significantly correlate.

Thus, hypothesis 1 is confirmed.

To answer hypothesis 2, we calculated mean scores for competence and warmth and compared these with each other. The within subjects/paired samples *t*-test showed that competence scores were higher than those of warmth: *M* = 3.84, *SD* = 0.61 for competence and *M* = 3.48, *SD* = 0.66 for warmth; *t*_(239)_ = 9.38, *p* < 0.001. These two stereotypes are moderately correlated: *r* = 0.56, *p* < 0.001.

Hypothesis 2 is confirmed.

To address hypothesis 3, we calculated the English-speaking group's mean scores for nine emotions and ANOVA to compare them, see Table 2:

**Table 2:** Mean scores of emotions for the English-speaking group.

	**Admiration**	**Sympathy**	**Envy**	**Pride**	**Anger**	**Resentment**	**Pity**	**Contempt**	**Disgust**
English-speaking group	**3.32 (0.95)**	**3.24 (0.93)**	**3.07 (1.20)**	**2.97 (0.92)**	2.60 (1.03)	2.51 (1.11)	2.45 (1.13)	2.37 (1.04)	2.26 (1.04)

*Standard deviations are shown in parentheses*.

*Scores that significantly differ from the other scores are provided in bold*.

As in the case of the German-speaking group, four emotions can be regarded as having high scores, which sets them apart from all other emotions, *F*_(8, 1, 784)_ = 37.06, *p* < 0.001. At the same time, according to pairwise comparisons, envy is not different from pride, sympathy, and admiration.

To sum up, for the English-speaking group, hypotheses 1 and 2 are confirmed, while hypothesis 3 was partially confirmed, as sympathy and envy gained high scores in addition to admiration and pride.

Next, prior to testing hypotheses 4 and 5, we proceeded with testing the correlations of vitality and fear of assimilation with warmth, competence, and nine emotions for the German- and English-speaking groups. The correlation coefficients and significance levels are provided in Table 3. The results below are presented separately for the German- and English-speaking groups.

**Table 3:** Correlations.

	**1**	**2**	**3**	**4**	**5**	**6**	**7**	**8**	**9**	**10**	**11**	**12**	**13**
Competence	1	0.40^***^	0.28	0.00	−0.19	−0.17	−0.25	−0.39^***^	−0.23	0.24	0.20	0.17	−0.24
Warmth	0.56^***^	1	0.15	−0.10	0.16	−0.17	−0.19	−0.27	−0.23	0.42^***^	0.17	0.24	−0.11
Vitality	0.27^***^	0.11	1	0.00	−0.08	0.10	−0.29^*^	0.03	−0.19	0.24	0.16	0.15	0.06
Fear of assimilation	−0.07	−0.08	−0.08	1	−0.10	0.03	0.21	0.14	0.30^*^	0.07	0.10	−0.02	0.17
Pity	0.05	0.14	−0.00	−0.01	1	0.00	0.09	0.15	0.11	0.22	0.23	0.09	0.28
Envy	0.09	−0.11	0.24^***^	0.10	−0.00	1	0.19	0.41^***^	0.10	0.02	−0.08	0.25	0.24
Contempt	−0.15	−0.14	0.10	0.07	0.11	0.23^***^	1	0.34^*^	0.29^*^	−0.19	−0.17	−0.07	0.25
Anger	−0.19^*^	−0.35^***^	0.13	0.08	0.04	0.22^**^	0.41^***^	1	0.52^***^	−0.03	0.12	0.07	0.67^***^
Resentment	−0.26^**^	−0.36^***^	0.02	0.09	−0.06	0.26^***^	0.39^***^	0.65^***^	1	−0.13	0.09	−0.24	0.52^***^
Pride	0.28^***^	0.37^***^	0.09	0.05	0.09	0.01	−0.09	−0.15	−0.19^*^	1	0.22	0.49^***^	0.02
Sympathy	0.26^***^	0.46^***^	−0.02	0.05	0.24^***^	−0.05	−0.13	−0.20^**^	−0.22^**^	0.41^***^	1	0.33^*^	0.19
Admiration	0.33^***^	0.36^***^	0.14	0.01	0.07	0.13	−0.25^***^	−0.18^*^	−0.23^***^	0.47^***^	0.41^***^	1	0.10
Disgust	−0.24^***^	−0.30^***^	−0.05	0.08	−0.04	0.19^**^	0.36^***^	0.48^***^	0.49^***^	−0.04	−0.24^***^	−0.19^**^	1

*** *p < 0.001*;

** *p < 0.01*;

* *p < 0.05*.

*Correlations for the German-speaking group are provided in the upper diagonal, while correlations for the English-speaking group are represented in the lower diagonal*.

#### German-Speaking Group

To test hypothesis 4 that vitality and fear of assimilation predict corresponding stereotypes independently as well as in interaction with each other, we ran regression analysis through entering gender and age in the first model and vitality and fear of assimilation in the second model and then moved to PROCESS MACRO to examine interaction terms. In the case of the German-speaking group, vitality predicted competence, β = 0.33, *p* < 0.05. No significant interaction was found.

To examine whether stereotypes and social–structural antecedents predict the corresponding emotions independently, as well as in interaction with one other (hypothesis 5), we regressed each of the nine emotions on vitality, fear of assimilation, competence, and warmth scores in a two-model way (with vitality, fear of assimilation, warmth, and competence scores included in the second model) and moved to the PROCESS MACRO to examine interaction terms. For the German-speaking group, out of nine emotions, six were predicted by some of the four variables. Either competence or warmth predicted pride, admiration, and sympathy. In addition, competence was a negative predictor of anger and resentment, and age was a negative predictor of sympathy. Also, vitality predicted contempt (negatively), while fear of assimilation predicted sympathy and resentment (Table 4). No interaction term was significant in moderation analysis (PROCESS MACRO).

**Table 4:** Regression analysis: predictors of emotions for the German-speaking group.

	**Model**			**Coefficients**	
	**Δ*R***^**2**^	**Sig. *F* change**	***β***	***t***	**Sig**.
**Predictors for pride**	0.22	0.006			
Warmth			0.36	3.01	0.014
**Predictors for admiration**	0.29	0.000			
Competence			0.47	4.10	0.000
**Predictors for sympathy**	0.16	0.014			
Competence			0.24	2.00	0.050
Fear of assimilation			0.29	2.68	0.014
Age			−0.31	−2.80	0.014
**Predictors for anger**	0.19	0.014			
Competence			−0.29	−2.20	0.035
**Predictors for resentment**	0.23	0.006			
Competence			−0.31	−2.38	0.027
**Predictor for Contempt**	0.19	0.014			
Vitality			−0.34	−2.68	0.014

Next, mediation analysis was conducted to address hypotheses 6 and 7 but did not yield any significant results. Thus, hypotheses 6 and 7 were rejected for the German-speaking group.

To sum up, for the German-speaking group, hypotheses 4, 6, and 7 were rejected, while hypothesis 5 was supported partially.

#### English-Speaking Group

To test hypothesis 4, we ran a similar regression analysis as mentioned above. Vitality predicted competence positively, β = 0.25, *p* < 0.01, explaining 8% of variance in competence scores. Vitality and fear of assimilation did not interact.

Then, we tested hypothesis 5 for the English-speaking group in a similar way to the German-speaking group. For the English-speaking group, admiration and envy were predicted by warmth and competence; also, vitality was a positive predictor of envy; anger was predicted by vitality and warmth. Pity was not predicted at all, and the rest of the emotions were predicted by one of the predictors only—contempt by competence (negatively), pride and sympathy by warmth, resentment and disgust by warmth (negatively) (Table 5).

**Table 5:** Regression analysis: predictors of emotions for the English-speaking group.

	**Model**			**Coefficients**	
	**Δ*R***^**2**^	**Sig. *F* change**	***β***	***t***	**Sig**.
**Predictors for pride**	0.13	0.000			
Warmth			0.29	3.47	0.003
**Predictors for admiration**	0.16	0.000			
Competence			0.18	2.16	0.035
Warmth			0.26	3.18	0.004
**Predictors for sympathy**	0.27	0.000			
Warmth			0.50	6.44	0.000
**Predictors for anger**	0.19	0.000			
Warmth			−0.39	−4.79	0.000
Vitality			0.25	3.53	0.024
**Predictors for resentment**	0.15	0.000			
Warmth			−0.32	−3.81	0.000
**Predictors for disgust**	0.08	0.003			
Warmth			−0.21	−2.44	0.024
**Predictors for contempt**	0.080	0.004			
Competence			−0.20	−2.26	0.030
**Predictors for envy**	0.08	0.003			
Competence			0.18	2.01	0.046
Warmth			−0.20	−2.29	0.030
Vitality			0.19	2.56	0.018

**Figure 1:**
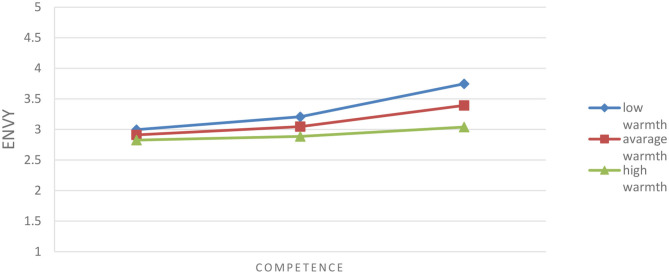
Effect of interaction of competence and warmth on envy.

The moderation analysis in PROCESS MACRO yielded significant interaction of competence and warmth in the case of envy: *F*_(1, 177)_ = 4.13, *p* < 0.05, Δ*R*^2^ = 0.02. Figure 1 below shows that competence has an effect on envy, namely, increases it when warmth is low (1 SD below the mean) and moderate (the mean), while the effect disappears (is not significant) when warmth is high (1 SD above the mean).

**Figure 2:**
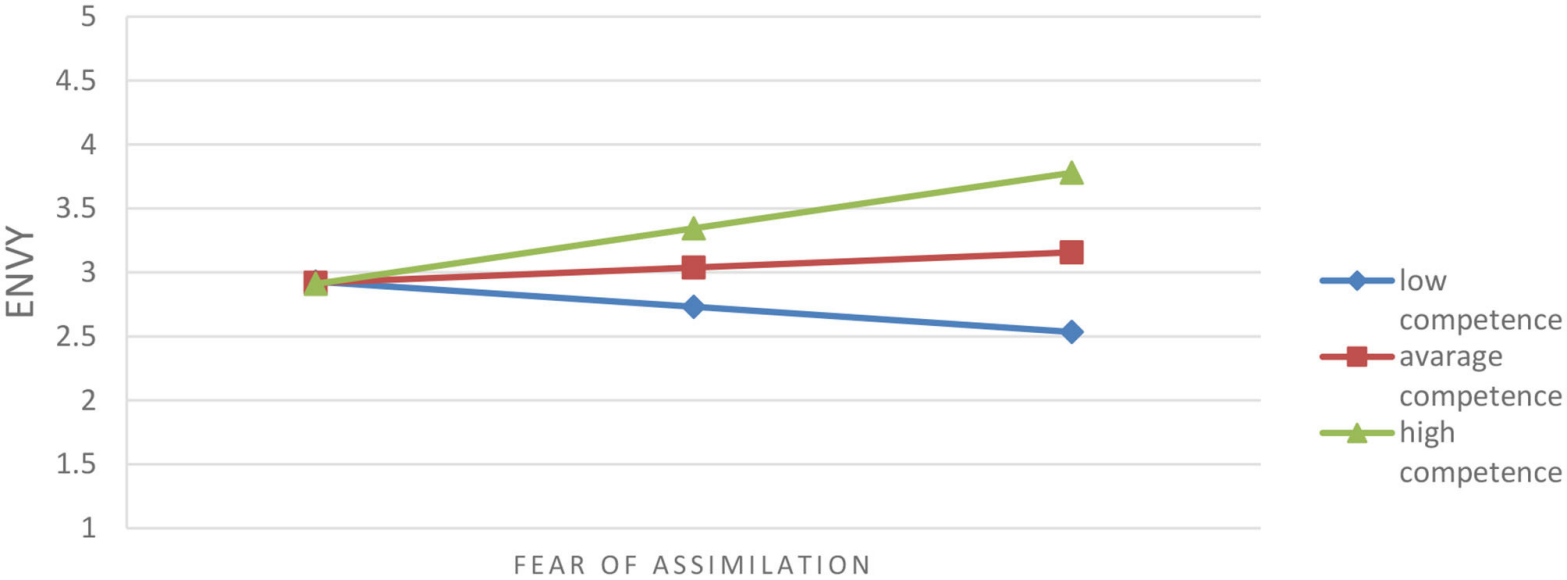
Effect of interaction of fear of assimilation and competence on envy.

Another moderation analysis detected an interaction of fear of assimilation and competence in case of envy. The interaction model is significant, *F*_(1, 177)_ = 10.35, *p* < 0.01, Δ*R*^2^ = 0.05. As we can see in Figure 2, fear of assimilation has an effect on envy when competence is high, while it does not have an effect on envy (it is not statistically significant) when competence is moderate or low. In other words, we can say that the emotion of envy is predicted not only because of competence ascribed to the English-speaking group but also because of fear of assimilation.

**Figure 3:**
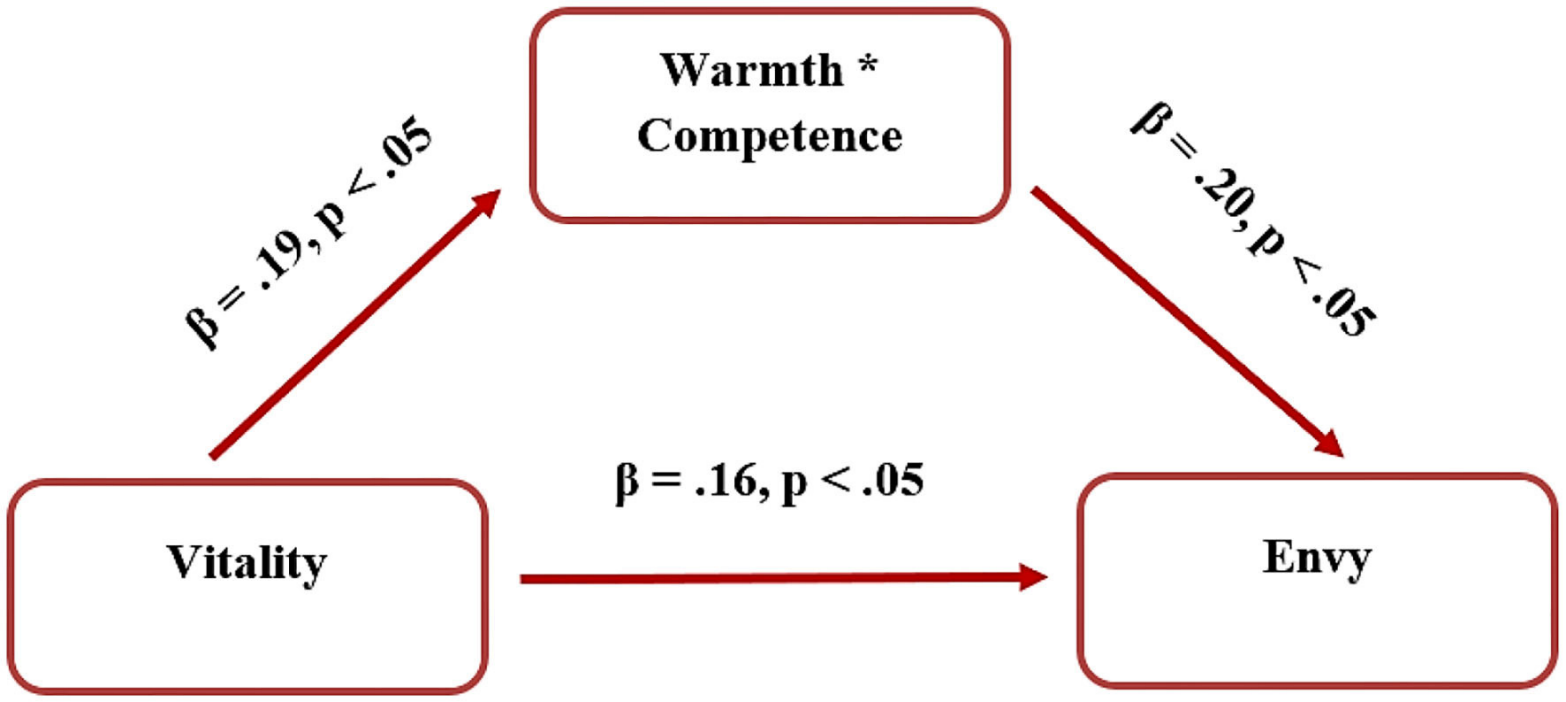
Vitality as a predictor of envy, mediated by the interaction of warmth and competence.

Next, mediation analysis was conducted to address hypotheses 6 and 7 for the English-speaking group. For the English-speaking group, regression analysis showed that vitality directly predicted both competence and envy. Thus, we were able to test the mediation model, where vitality predicted envy mediated by competence, but it was not significant. However, a more refined mediation model of vitality predicting envy through the interaction of warmth and competence (described above) as a mediator was significant (see Figure 3). The indirect effect of vitality on envy is: *b* = 0.09, lower level confidence interval (LLCI) = 0.01–upper level confidence interval (ULCI) = 0.20. The total effect of vitality on envy is 0.45, which consists of the direct effect 0.36 and indirect effect through the mediator 0.09.

To sum up, for the English-speaking group, hypothesis 4 was partially supported, hypothesis 5 was supported, hypothesis 6 was rejected, while hypothesis 7 was partially supported.

## Discussion

We examined the role of the two social–structural antecedents in eliciting stereotypes and emotions independently as well as in interaction with each other and the role of the two stereotypes in eliciting emotions independently as well as in interaction with each other. The design of our study enabled us to address more closely each member of this chain. The multiple findings of the study speak to the SCM and beyond it, to the theory of prejudice as well as touch upon understanding of emotions.

### The Intergroup Emotions

The finding that the perceived stereotypes elicit emotions of admiration and pride, but, also, they elicit sympathy toward German- and English-speaking group representatives, deviates from the SCM, according to which sympathy should not be paired either with admiration and pride or with anger and envy. This finding can be explained by the Georgian respondents' understanding of its meaning. The translation of emotion-defining adjectives was a rather difficult process, and we had to check and double-check their meanings with one of the authors of the model, Susan Fiske. The term “sympathy” was translated effortlessly, as it has an equivalent in Georgian. However, after this unexpected finding, we conducted a small expert-type study with our linguist and psychologist colleagues and found out that the Georgian equivalent of “sympathy” can rather be understood as “empathy,” which means that, in our case, the out-groups' perspective and emotions are understood. Indeed, when measuring “sympathy,” one of the studies also employed emotions of “empathy” and “compassion” (Sweetman et al., 2013). We demonstrated the two factorial structures of stereotype scale *via* CFA, an approach that can be rarely seen in other original versions of the SCM scale (Durante, 2008; Stanciu et al., 2017; Kotzur et al., 2018, 2019), thus validating the Georgian version of the instrument; however, the translation proved to be a challenge because we had to drop the 10th emotion, “jealousy.”

Also, interestingly enough, admiration was predicted by competence only in the case of the German-speaking group and by both competence and warmth in the case of the English-speaking group. The former finding coincides with that of the 2013 study (Sweetman et al., 2013) and the latter with those of Fiske et al. (2002) and Cuddy et al. (2008). Emotional theorists consider admiration as containing both competence and moral aspects, which is connected to warmth in the SCM. Thus, admiration is connected with both warmth and competence. Indeed, in one of the studies (Sweetman et al., 2013), admiration was measured by such items as “respect” as well (study 4), pointing to its moral component. One of the possible explanations of why the groups in question deserved such positive emotions can be entitativity, which characterizes homogeneous, organized groups with shared goals. The authors found that entitativity affects warmth stereotype perception through increasing it (Dang et al., 2018). Thus, it could be argued that our participants perceived the German- and English-speaking groups as entitative.

These findings were possible because of two reasons: firstly, we studied single emotions and did not group them as it is usually done (Fiske et al., 2002; Cuddy et al., 2007; Bye and Herrebrøden, 2018); and secondly, we regressed these emotions on competence and warmth to see which of them are positively predicted by stereotypes. Analyzing single emotions separately enabled us to look deeper into the nature of emotions on the one hand and into the links between stereotypes and emotions on the other. We assigned emotions to stereotypes *via* regression analysis that can also be used in addition to calculating means and comparing them, especially if we analyze single emotions. Means tell us which emotions are felt the most, while regression tells us which emotions are linked with stereotypes. The regression analysis has been used in a number of studies using status and competition as predictors of warmth and competence (Durante, 2008; Kervyn et al., 2015; Grigoryev et al., 2019), while we have applied this approach to better investigate the links with emotions. Although in essence, regression and ANOVA are the same analyses, they make us look at the data and interpret them from different perspectives. Thus, we can conclude that single-sample/single-group design findings concerning emotions conducted on an individual-level analysis enriched our understanding of how they are elicited.

### Stereotype Content Model and Social–Structural Antecedents

Below we will analyze our findings starting from the social–structural antecedents ending with the elicited emotions *via* stereotypes, following the SCM logic. In terms of the social–structural antecedents, fear of assimilation does not predict warmth, while vitality predicts competence (hypothesis 4). The study of different immigrant groups in the Unites States found the same connections: while group-level analysis revealed both links, individual-level analysis, as in our case, could only confirm the status/competence link (Lee and Fiske, 2006). Also, vitality predicts envy, while fear of assimilation does not and neither does their interaction. Study of nine post-socialist bloc societies found that the link between competition and warmth is higher in these societies than in the capitalist countries (Grigoryan et al., 2019). The possible explanation for our case is that our proxy measure of competition, fear of assimilation, contained questions on symbolic threat but not on realistic threat. Also, Durante (2008) proposed to consider cooperation as a better predictor of warmth.

The effect of competence on envy is conditioned by warmth and *vice versa* (hypothesis 5). The combination of competence and warmth is needed to elicit envy, but if one of these stereotypes is high enough, the second one is not needed. Only in two studies (Sweetman et al., 2013; Kotzur et al., 2018) could we find a similar idea of checking the warmth and competence interaction to predict emotions. No interaction of warmth and competence was found to be significantly linked with admiration, pity, and contempt in the 2013 study, while pity, contempt, and envy were predicted by interaction of warmth and competence scores in the 2018 study. We found that envy is elicited not only by competence and warmth but also by the interaction of competence with fear of assimilation (hypothesis 5). The increase of fear of assimilation is linked with an increase in envy, but only when the level of perceived competence is moderate or high. This finding, on the one hand, corresponds to the SCM postulate that stereotypes and their antecedents predict emotions but, on the other hand, deviates from the SCM logic that competition interacts with competence and not warmth. The study of stereotypes and their antecedents in Russia has also found that perception of economic threat is linked with competence (Grigoryev et al., 2019). Fear of assimilation is the same as perceived symbolic threat. Indeed, the ITT posits that threat, a situational variable, is needed to increase prejudice (Stephan et al., 2009). Previous studies conducted in Georgia found the same (Makashvili, 2018; Makashvili et al., 2018).

In addition, vitality is also connected to envy directly as well as *via* the mediation of warmth and competence product term (hypothesis 7). According to the SCM framework, perceived social–structural antecedents elicit stereotypes, which in turn elicit emotions. This chain from antecedents to stereotypes and emotions has not been demonstrated in the literature so far, and our mediational chain enables us to clearly show how the emotion of envy is elicited. The envy predicted toward the representatives of the English-speaking group might also be explained by the nature of envy itself: as the authors of the BIAS map note, envy is an ambivalent emotion, involving respect and resentment at the same time, while an “ambivalent type of respect is […] a begrudging admiration for the other” (Cuddy et al., 2007, p. 634). Furthermore, Norwegian authors distinguish between two types of envy: malicious and non-malicious. The former is close to the feelings of anger and resentment, while the latter is close to the feeling of admiration, which is also felt toward the English-speaking group in our study. With this nature of envy, they explain the finding of only envy mediating relation between competence and the corresponding behavior, assuming that their participants experienced non-malicious envy (Bye and Herrebrøden, 2018). In the study of the mental illness stigma, admiration and envy loaded on one factor (Sadler et al., 2015). Following this reasoning, and considering that our respondents make an upward social comparison with the representatives of the English-speaking group, we may also assume that our participants envy the representatives of English-speaking countries in a non-malicious way. Thus, applying moderation models enabled us to demonstrate that interaction of stereotypes, as well as perceived status and competition predict emotions.

### Application of Stereotype Content Model in Georgia

Finally, our findings provide a deeper insight into how Georgian students view representatives of the German- and English-speaking countries. They confirm findings of our previous studies where these groups are held close to Georgian students in terms of social distance (Javakhishvili, 2005; Javakhishvili et al., 2018). Indeed, representatives of both groups are perceived as having high status and being less competitive, also, competent and warm, deserving emotions of pride, admiration, and sympathy, which propose explanation to why they are held so close. English- and German-speaking countries—the USA, UK, Germany, etc.—are highly developed, powerful nations that play an important role in the international arena. English is the main foreign language in Georgia as well as elsewhere; the knowledge of the English language is required to get a good job. Germany is also a powerful country supporting Georgia; however, less Georgians speak German than English. For Georgians, the USA and the European Union are especially important, as they support the country's democratic development and its unstable economy.

Envy is also felt toward the English-speaking group representatives, as demonstrated by different data processing techniques, including mediational chain from vitality through warmth and competence interaction. Georgians consider English-speaking people as highly competent and warm, but, at the same time, as a threat to the Georgian language and traditions—in other words, as a source of symbolic threat, as defined by the ITT (Stephan et al., 2009). Symbolic threat is represented by fear of assimilation in our study, which in interaction with competence elicited envy. The prediction of envy is explained by moderation analysis, according to which it is elicited by the interplay of warmth and competence, as well as fear of assimilation and competence. Envy itself can be understood 2-fold: as non-malicious or malicious, the former, in our view, being the participants' emotion to the English-speaking group. Thus, such emotion does not prevent our participants from holding this out-group close. In sum, distant out-groups elicit envy as a result of their perceived vitality, fear of assimilation, warmth, and competence.

## Limitations and Future Research

One of the limitations of this study is a single-group design, which does not allow for cluster solution of data and variability. Divergence in measures is also to be seen as the study limitation: one such divergence was related to the corresponding set of emotions, as mentioned, we had to drop one (jealousy) and the other (sympathy) was understood differently from the original version. Another divergence was related to social–structural antecedents: we used substitute variables instead of applying the original questions of competition and status. This substitution, nevertheless, was justified by the specifics of the out-group studied and enabled us to detect certain links. However, lack of items tapping into realistic threat indeed created an obstacle. The study of group threat perceptions and emotions points that symbolic and realistic threats might elicit different emotions because of the different nature of the threats as well as emotions (Matthews and Levin, 2012). Therefore, having realistic threat items at hand would have given us more important information about these links. Further research might be envisaged with more out-groups to add variability to the data. Indeed, we have already planned a new study, where we will use a more precise translation of emotions as well as eight out-groups to be assessed, which in our view will provide enough variability to gain four stereotype clusters. Also, the future study will incorporate an expanded measure of the scale of competition and status by Fiske et al., so that cooperation is also included in investigating links between social–structural antecedents and stereotypes.

## Conclusions

The added value of this research should be considered in two directions: firstly, it contributes to the SCM theory, which works differently when small country representatives evaluate representatives of large and powerful countries. When analyzing emotions separately, and *via* regression analysis, more than two emotions are elicited. We have demonstrated that the link from perceived vitality to envy is mediated by interaction of warmth and competence. Such mediation has not been examined until now. Also, we were able to demonstrate for the first time that perceived competitiveness/fear of assimilation interacts with competence to predict envy. Secondly, this research contributes to the general theory of prejudice measured by social distance to geographically distant out-groups—findings of our previous studies that English- and German-speaking people have consistently been held close can be explained by the SCM. German-speaking group representatives are considered competent and warm, thus eliciting admiration and pride, which apparently can explain short social distance. English-speaking group representatives, in addition, elicit the emotion of envy, as demonstrated by mediational chain from vitality through warmth and competence interaction. Social–structural antecedents come forward to elicit emotion of envy independently as well as in interaction with stereotypes when small country representatives evaluate representatives of the influential group of English-speaking people.

## Data Availability Statement

The raw data supporting the conclusions of this article will be made available by the authors, without undue reservation.

## Ethics Statement

The studies involving human participants were reviewed and approved by Ilia State University Ethics Committee. The patients/participants provided their written informed consent to participate in this study.

## Author Contributions

NJ was the principal investigator of the project, coordinated all the processes starting from the inception phase of formulating research questions through data collection, processing and analyses, and ending with writing up the research. NB contributed to organizing the database, participated in data processing, and wrote the Methods section of the paper. IV participated in the data processing and analysis and writing up the research. AG proposed the overall idea of the study and collected the corresponding data. All authors have contributed to manuscript revision, read, and approved the submitted version.

## Conflict of Interest

The authors declare that the research was conducted in the absence of any commercial or financial relationships that could be construed as a potential conflict of interest.
